# Nonlinear versus Ordinary Adaptive Control of Continuous Stirred-Tank Reactor

**DOI:** 10.1155/2015/389273

**Published:** 2015-08-04

**Authors:** Jiri Vojtesek, Petr Dostal

**Affiliations:** Faculty of Applied Informatics, Tomas Bata University in Zlin, Nam. T.G. Masaryka 5555, 760 01 Zlin, Czech Republic

## Abstract

Unfortunately, the major group of the systems in industry has nonlinear behavior and control of such processes with conventional control approaches with fixed parameters causes problems and suboptimal or unstable control results. An adaptive control is one way to how we can cope with nonlinearity of the system. This contribution compares classic adaptive control and its modification with Wiener system. This configuration divides nonlinear controller into the dynamic linear part and the static nonlinear part. The dynamic linear part is constructed with the use of polynomial synthesis together with the pole-placement method and the spectral factorization. The static nonlinear part uses static analysis of the controlled plant for introducing the mathematical nonlinear description of the relation between the controlled output and the change of the control input. Proposed controller is tested by the simulations on the mathematical model of the continuous stirred-tank reactor with cooling in the jacket as a typical nonlinear system.

## 1. Introduction

The control of the chemical processes in the industry is always challenging because of the nonlinearity of the major group of systems. The continuous stirred-tank reactor (CSTR) is one of the most common used types of chemical reactors because of easy controllability [1].

The adaptive control [2] is a control technique with good theoretical background and also practical implementations. It uses idea of the living organisms that adopts their behavior to the actual environmental conditions. There are also various adaptation techniques and variations described, for example, in [3].

The control method used here is based on the combination of the adaptive control and nonlinear control. Theory of nonlinear control (NC) can be found, for example, in [4, 5]. The nonlinear adaptive controller is divided via Wiener's model [6] into two parts: the dynamic linear part (DLP) and the static nonlinear part (SNP). The DLP uses polynomial synthesis [7] with pole-placement method and spectral factorization and all these methods satisfy basic control requirements such as disturbance attenuation, stability, and reference signal tracking. The second, nonlinear, part uses measurements of the steady-state behavior of the system for mathematical description of the dependence between the controlled output variable and the control input variable.

The controlled system, CSTR, with originally nonlinear behavior could be mathematically described for the control purposes by the external linear model (ELM) [8], parameters of which could vary because of the nonlinearity of the system. This problem could be overcome with the use of recursive identification which recomputes parameters of the ELM according to the actual state and the behavior of the system. There were used delta- (*δ*-) model [8] in this work as a special type of discrete-time models parameters of which approaches to the continuous ones for the small sampling period as it is proofed, for example, in [9].

The results are also compared with classical adaptive control which uses only ELM as a linear representation of the originally nonlinear controller [10, 11] to show the improvement of this nonlinear adaptive control strategy.

The proposed control strategies were verified by simulations on the mathematical model of CSTR with cooling in the jacket [12]. This mathematical model was studied also in [10] and classic adaptive controller was applied in [11]. All simulations were done in the mathematical software Matlab, version 7.0.1.

## 2. Controlled Plant

**Figure 1:**
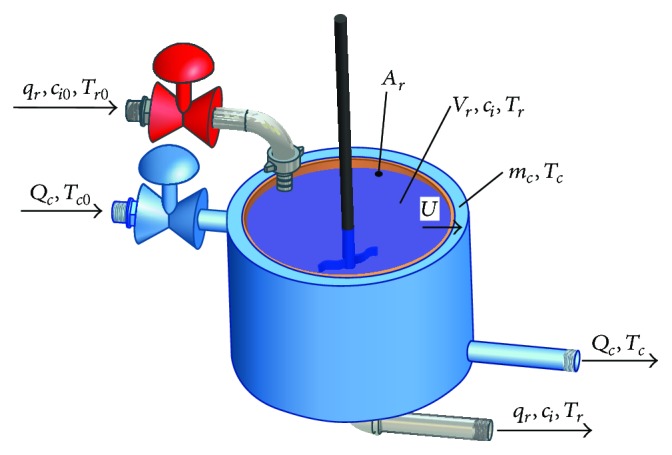
Continuous stirred-tank reactor with cooling in the jacket.

The system under the consideration is a continuous stirred-tank reactor (CSTR) with the so-called* Van der Vusse reaction A* → *B* → *C*, 2*A* → *D* inside and cooling jacket—see the scheme of the CSTR in Figure 1.

If we introduce common simplifications like the perfect mixture of the reactant, all densities, transfer coefficients, heat capacities, and the volume of the reactant are constant throughout the reaction, and the mathematical model developed with the use of material and heat balances inside has form of the set of ordinary differential equations (ODEs) [12]

(1) dcAdt=qrVrcA0−cA−k1cA−k3cA2,dcBdt=−qrVrcB+k1cA−k2cB,dTrdt=qrVrTr0−Tr−hrρrcpr+ArUVrρrcprTc−Tr,dTcdt=1mccpcQc+ArUTr−Tc,

where *t* in (1) is the time, *c* are concentrations, *T* represents temperatures, *c*_*p*_ is used for specific heat capacities, *q*_*r*_ means the volumetric flow rate of the reactant, *Q*_*c*_ is the heat removal of the cooling liquid, *V*_*r*_ is volume of the reactant, *ρ* stands for densities, *A*_*r*_ is the heat exchange surface, and *U* is the heat transfer coefficient. Indexes (·)_*A*_ and (·)_*B*_ belong to compounds *A* and *B*, respectively, (·)_*r*_ denotes the reactant mixture, (·)_*c*_ denotes cooling liquid, and (·)_0_ are feed (inlet) values.

The variable *h*_*r*_ and *k*_1−3_ in (1) denote the reaction heat and reaction rates which are computed from

(2) hr=h1·k1·cA+h2·k2·cB+h3·k3·cA2,kjTr=k0j·exp−EjRTr, for  j=1,2,3,

where *h*_*i*_ stands for reaction enthalpies. Reaction rates *k*_1−3_ in the second equation are nonlinear functions of the reactants temperature computed via* Arrhenius law* with *k*_0*j*_ as rate constants, *E*_*j*_ are activation energies, and *R* means gas constant.

Equations (1) together with (2) construct the* mathematical model of the plant* used later for simulation studies. Due to simplifications introduced above we can say that this type of reactor is* a nonlinear lumped-parameters system*. We have four state variables *c*_*A*_, *c*_*B*_, *T*_*r*_, and *T*_*c*_ and four input variables: the volumetric flow rate of the reactant, *q*_*r*_, the heat removal of the coolant, *Q*_*c*_, the input concentration, *c*_*A*0_, and input temperature of the reactant, *T*_*r*0_. The fixed values of the reactor are shown in Table 1 [12].

It is good to know behavior of the system before the design of the controller. This behavior is usually obtained from the steady-state and dynamic analyses of the system which will be described in the next subchapters.

### 2.1. Steady-State Analysis

This analysis observes the behavior of the system in the steady-state, that is, in time *t* → *∞*. Mathematically speaking, derivatives with respect to time in the set of ODEs (1) are equal to zero; that is,

(3) $$\begin{array}{c} \frac{d\left(\cdot \right)}{dt}=0 \end{array}$$

which means that the set of ODEs (1) is transformed to the set of nonlinear algebraic equations that can be solved, for example, with the simple iterative method. This method is easily programmable in common mathematical software.

**Figure 2:**
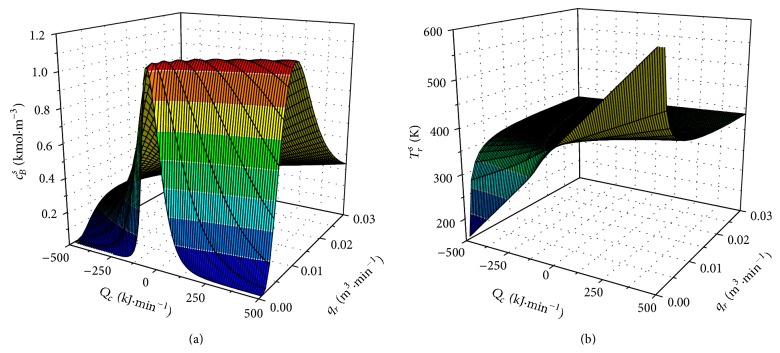
Steady-state characteristics of the product's concentration *c*_*B*_^*s*^ (a) and reactant's temperature *T*_*r*_^*s*^ (b).

Results of steady-state analyses for different volumetric flow rate of the reactant *q*_*r*_ = 〈0; 0.03〉  m^3^ · min^−1^ and heat removal of cooling *Q*_*c*_ = 〈−500; 500〉  kJ · min^−1^ are shown in Figures 2(a) and 2(b).

Both graphs show highly nonlinear steady-state behavior of this system.

### 2.2. Dynamic Analysis

**Figure 3:**
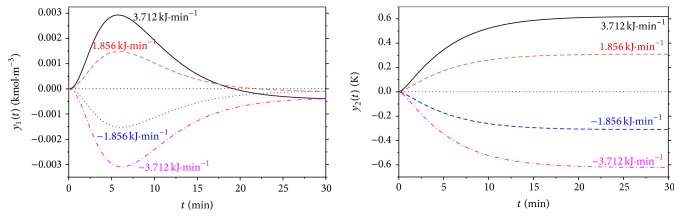
Results of dynamic analysis for the step changes of the heat removal of the cooling, Δ*Q*_*c*_.

**Figure 4:**
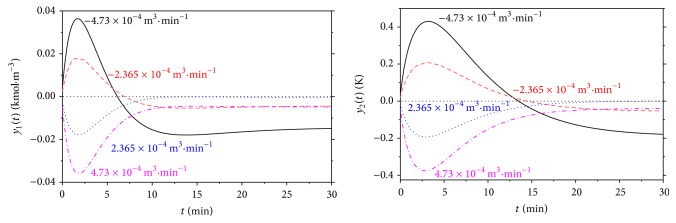
Results of dynamic analysis for the step changes of the volumetric flow rate of the reactant, Δ*q*_*r*_.

The second, dynamic, analysis shows the response of the system to the step change of the input quantity. Although there could be theoretically four input quantities, the volumetric flow rate of the reactant, *q*_*r*_, and heat removal of the cooling, *Q*_*c*_, were chosen as an input variables mainly from the practical point of view. Figures 3 and 4 show dynamic responses for various step changes of the input quantities in the working point *q*_*r*_^*s*^ = 2.365 · 10^−3^ m^3^ · min^−1^ and *Q*_*c*_^*s*^ = −18.56 kJ · min^−1^. Inputs *u*_1_ and *u*_2_ represent step changes of the *q*_*r*_ and *Q*_*c*_, respectively, and outputs *y*_1_ and *y*_2_ show difference of the output products concentration, *c*_*B*_, and reactants temperature, *T*_*r*_, from their initial, that is, steady-state, value:

(4) u1t=Qct−Qcs  kJ·min−1,u2t=qrt−qrs  m3·min−1,y1t=cBt−cBs  kmol·m−3,y2(t)=Tr(t)−Trs  K,

where initial values of *c*_*B*_ and *T*_*r*_ are *c*_*B*_^*s*^ = 1.0903 kmol · m^−3^ and *T*_*r*_^*s*^ = 387.34 K.

## 3. Nonlinear Adaptive Control Strategy

**Figure 5:**
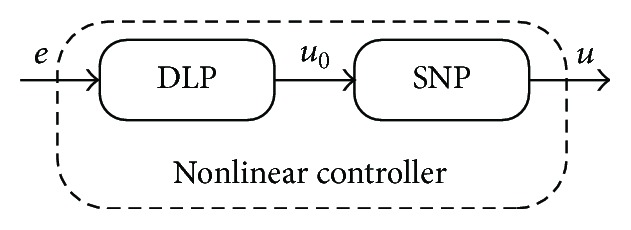
The scheme of the nonlinear controller.

The control strategy here is based on the factorization of controller into the static nonlinear part (SNP) and the dynamic linear part (DLP); see Figure 5. This control scheme configuration is called a* Wiener system*.

As written in the previous part, there are theoretically four input and four output variables. In this case, the change of the output concentration, *c*_*B*_, from its steady-state value, *c*_*B*_^*s*^, was controlled with the change of the volumetric flow rate of the reactant, *q*_*r*_, from the working point, *q*_*r*_^*s*^; that is,

(5) ut=Δqr=qrt−qrsm3·min−1,yt=ΔcB=cBt−cBskmol·m−3.

The dynamic part DLP in Figure 5 represents linear dynamic relation between the tracking error *e*(*t*) and the input to the nonlinear static part *u*_0_(*t*) = Δ*c*_*Bw*_(*t*) which is difference between the concentration of the product, *c*_*B*_(*t*), and its desired value. The second static nonlinear part then describes the relation between *u*_0_(*t*) and corresponding change of the input volumetric flow rate of the reactant Δ*q*_*r*_(*t*).

**Figure 6:**
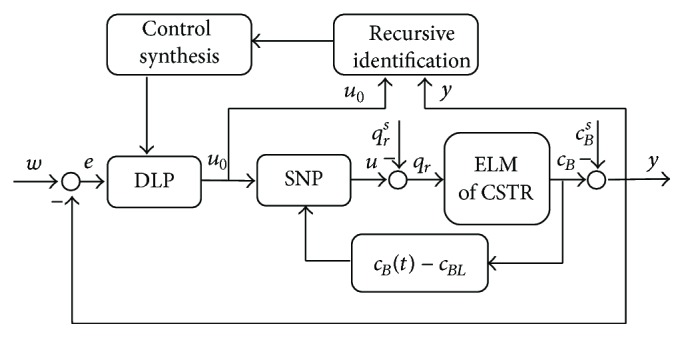
Control scheme of the nonlinear adaptive control.

The schematic representation of the control system can be found in Figure 6.

### 3.1. Static Nonlinear Part

The nonlinear part uses properties of the system in the steady-state described above.

**Figure 7:**
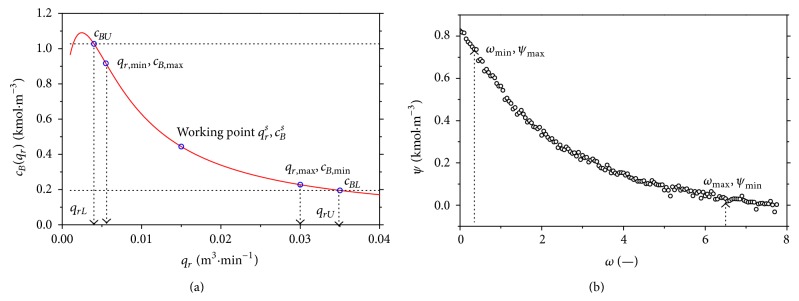
The steady-state characteristic (a) and noised data in new coordinates *ψ* = *f*(*ω*) (b).

If we do the steady-state characteristic for the volumetric flow rate of the reactant, *q*_*r*_, from the range *q*_*r*_ = 〈0.001; 0.04〉  m^3^ · min^−1^, results for the steady-state values of the products concentration, *c*_*B*_^*s*^, are shown in Figure 7(a). The operation of the controller was chosen in the interval where *q*_*r*,min_ = 0.0055 m^3^ · min^−1^ and *q*_*r*,max_ = 0.03 m^3^ · min^−1^. Working point of the system was chosen in the middle of this interval and includes also the nonlinearity of the system. This point is defined by the volumetric flow rate *q*_*r*_^*s*^ = 0.015 m^3^ · min^−1^ and heat removal of the coolant *Q*_*c*_^*s*^ = −18.56 kJ · min^−1^. The steady-state value of the controlled concentration is in this point *c*_*B*_^*s*^ = 0.442 kmol · m^−3^.

Due to later approximation and better unification of the variables, the new *x* and *y* variables *ω* and *ψ* are introduced and

(6) $$\begin{array}{c} \omega =\frac{q_{r}^{s}-q_{rL}}{q_{rL}}\left[\text{—}\right];\;\;\psi =c_{B}^{s}-c_{BL}\;\;\left[kmol\cdot m^{-3}\right], \end{array}$$

where *q*_*rL*_ is lower bound from the interval and *c*_*BL*_ is corresponding products concentration from the upper bound *q*_*rU*_; see Figure 7. It is recommended to choose this interval slightly longer than those in *q*_*r*,min_ ≤ *q*_*r*_(*t*) ≤ *q*_*r*,max_ which means in this case that lower and upper bounds of the input variable and equivalent values of the concentrations are

(7) qrL=0.004 m3·min−1,qrU=0.035 m3·min−1,cBL=0.1953 kmol·m−3,cBU=1.0274 kmol·m−3.

It is common that the measured data on the real system are affected by the measurement errors—see Figure 7(b) for new coordinates. To emulate these errors, the random white-noise error on the output variable is introduced here and the values for new coordinates with noised data are shown in Figure 7(b).

The difference of the input volumetric flow rate of the coolant is from (5)  *u*(*t*) = Δ*q*_*r*_(*t*) and the nonlinear part can be then computed from

(8) $$\begin{array}{c} u\left(t\right)=\Delta q_{r}\left(t\right)=q_{rL}{\left(\frac{d\omega }{d\psi }\right)}_{\psi \left(c_{B}\right)}u_{0}\left(t\right). \end{array}$$

The values of *q*_*rL*_ and *u*_0_(*t*) in (8) are known and the derivative *dω*/*dψ* is unknown.

**Figure 8:**
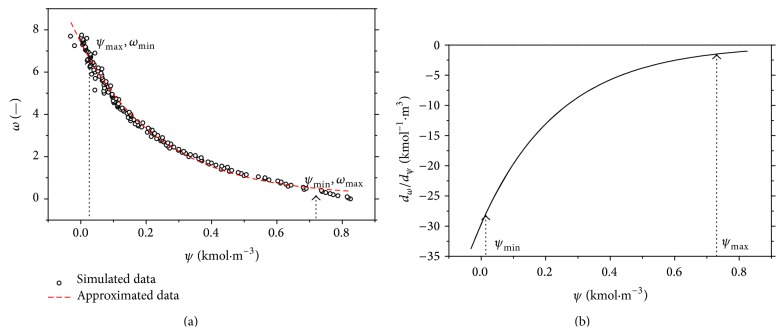
The simulated and approximated steady-state characteristic in new coordinates (a) and the course of the derivative of *dψ*/*dω* (b).

The procedure for computing of the value of this derivative for the specific value of products concentration, *c*_*B*_, is the following. The inverse of coordinates *ω* and *ψ* is done first; see Figure 8(a). Then, the data are approximated, for example, by the exponential, polynomial, and so forth, functions.

For example, the exponential function in the general form

(9) $$\begin{array}{c} \omega =f\left(\psi \right)=a\cdot e^{-b\cdot \psi }+c \end{array}$$

was used in this case. The course of this approximation is shown in Figure 8(a) (red dashed line) with the identified values of constants *a* = 7.1601, *b* = 4.1806, and *c* = 0.1707.

As there is the derivative *dω*/*dψ* in (8), this derivative is in this case

(10) $$\begin{array}{c} \frac{d\omega }{d\psi }=-29.9335\cdot e^{-4.1806\cdot \psi }. \end{array}$$

The course of this function is shown in Figure 8(b).

### 3.2. External Linear Model of CSTR

**Figure 9:**
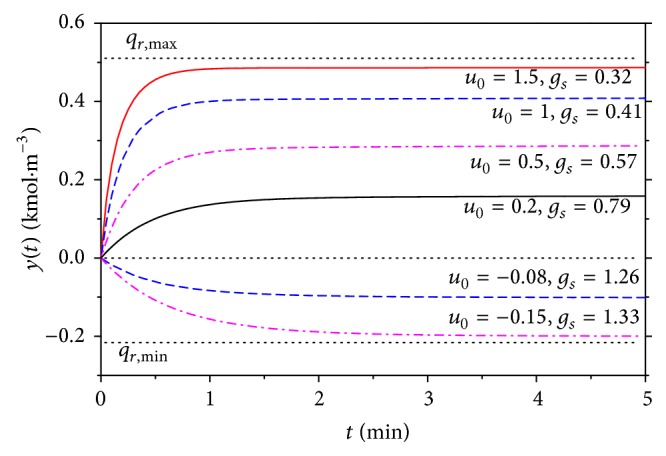
Results of dynamic analyses for the changes of input *u*_0_.

The dynamic behavior of the controlled system, in our case CSTR, together with the SNP derived above is observed for the step responses of the input *u*_0_; see Figure 2. Five changes *u*_0_ were done for the working point defined by input values *q*_*r*_^*s*^ = 0.015 m^3^ · min^−1^ and *Q*_*c*_^*s*^ = −18.56 kJ · min^−1^ and results are shown in Figure 9.

The gain of the system SNP+CSTR is computed as

(11) $$\begin{array}{c} g_{s}=\underset{t\to \infty }{lim}\frac{y\left(t\right)}{u_{0}} \end{array}$$

and the values of this gain, *g*_*s*_, are shown also in Figure 9.

Although the system has nonlinear behavior, presented output dynamic responses could be described by the first order continuous-time transfer function

(12) $$\begin{array}{c} G\left(s\right)=\frac{Y\left(s\right)}{U\left(s\right)}=\frac{b\left(s\right)}{a\left(s\right)}=\frac{b_{0}}{s+a_{0}} \end{array}$$

with *s* as a complex variable and polynomials *a*(*s*) and *b*(*s*) come from identification. This transfer function could be then in the form of the differential equation

(13) $$\begin{array}{c} \dot{y}\left(t\right)+a_{0}y\left(t\right)=b_{0}u\left(t\right). \end{array}$$

### 3.3. Identification of the ELM

The online identification of the continuous-time ELM (12) is not very simple. On the other hand, *δ*-identification models belong to the class of discrete models but their parameters are close to the continuous ones for very small sampling period.

The delta-model introduces a new complex variable *γ* as an alternative to complex variables *s* in continuous-time and *z* in discrete-time. The so-called forward *δ*-model for *β* = 0 was used here with the *γ* operator:

(14) $$\begin{array}{c} \gamma =\frac{z-1}{T_{v}}, \end{array}$$

where *T*_*v*_ is a sampling period and *z* is a discrete-time complex variable.

The continuous model (12) is then rewritten to the form

(15) $$\begin{array}{c} a^{\delta }\left(\delta \right)y\left(t^{\prime }\right)=b^{\delta }\left(\delta \right)u\left(t^{\prime }\right), \end{array}$$

where polynomials *a* ^*δ*^(*δ*) and *b* ^*δ*^(*δ*) are discrete polynomials and their coefficients are different from those of the CT models *a*(*s*) and *b*(*s*) in (12). Time *t*′ denotes discrete-time.

Equation (13) could be then with the substitution and simplifications rewritten to

(16) $$\begin{array}{c} y_{\delta }\left(k\right)=-a_{0}^{\delta }y_{\delta }\left(k-1\right)+b_{0}^{\delta }u_{\delta }\left(k-1\right), \end{array}$$

where new, recomputed, values of input and output variables are

(17) $$\begin{array}{c} y_{\delta }\left(k\right)=\frac{y\left(k\right)-y\left(k-1\right)}{T_{v}};\\ y_{\delta }\left(k-1\right)=y\left(k-1\right);\\ u_{\delta }\left(k-1\right)=u\left(k-1\right). \end{array}$$

The regression vector, *ϕ*_*δ*_, and vector of parameters, *θ*_*δ*_, used for identification are then

(18) φδ(k−1)=−yδ(k−1),uδ(k−1)T;θδk=a0δ,b0δT

and the differential equation (16) could be rewritten to the vector form:

(19) $$\begin{array}{c} y_{\delta }\left(k\right)=\theta_{\delta }^{T}\left(k\right)\cdot \varphi_{\delta }\left(k-1\right)+e\left(k\right), \end{array}$$

where *e*(*k*) is a general random immeasurable component. The task of the recursive identification is to find unknown vector of parameters, *θ*_*δ*_, from the measured data vector *ϕ*_*δ*_. The simple recursive least-squares (RLS) method was used in this work. This method together with exponential and directional forgetting modifications produces sufficient results as it was proofed by the previous experiments.

### 3.4. Dynamic Linear Part

The last part from Figure 6 which has not been discussed is the dynamic linear part (DLP). The feedback controller with one degree-of-freedom (1DOF) is designed with the use of polynomial approach [7].

**Figure 10:**
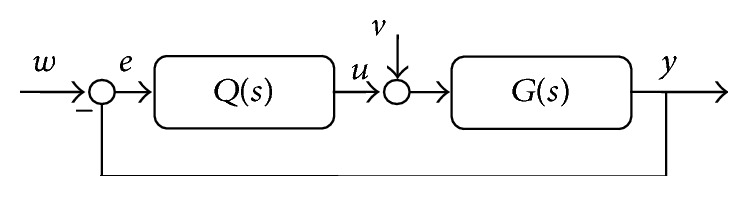
One degree-of-freedom (1DOF) control configuration.

The scheme of this control configuration is shown in Figure 10, where  *w* represents reference signal (wanted value), *e* is control error (*e* = *w* − *y*), *u* is control signal, *v* is immeasurable error, and *y* is controlled output from the system. The block *G*(*s*) is controlled system described by the transfer function (12) and *Q*(*s*) is feedback controller, the transfer function of which has general polynomial form:

(20) $$\begin{array}{c} \tilde{Q}\left(s\right)=\frac{q\left(s\right)}{s\cdot \tilde{p}\left(s\right)}, \end{array}$$

where parameters of polynomials and *q*(*s*) are computed from Diophantine equation:

(21) $$\begin{array}{c} a\left(s\right)\cdot s\cdot \tilde{p}\left(s\right)+b\left(s\right)\cdot q\left(s\right)=d\left(s\right). \end{array}$$

On the other hand, polynomials of the ELM *a*(*s*) and *b*(*s*) in (21) are known from the recursive identification and we expect that parameters of this polynomial are very close to parameters of correspondent *δ* polynomials *a* ^*δ*^(*δ*) and *b* ^*δ*^(*δ*) in (16). The polynomial *d*(*s*) on the right side of (21) is an optional stable polynomial and the degree of this polynomial is deg *d*(*s*) = deg *a*(*s*) + deg $\tilde{p}(s)$ + 1. Roots of this polynomial are called* poles of the closed-loop* and their position affects quality of the control.

There are several ways to construct this optional polynomial, for example, the pole-placement method, LQ approach, and so forth. The choice here combines the pole-placement method with spectral factorization of the identified polynomial *a*(*s*). The polynomial *d*(*s*) has then two parts:

(22) $$\begin{array}{c} d\left(s\right)=n\left(s\right)\cdot {\left(s+\alpha \right)}^{2}, \end{array}$$

where *α* > 0 is an optional coefficient reflecting closed-loop poles and stable polynomial *n*(*s*) is obtained from the spectral factorization of the polynomial *a*(*s*)—*n* ^⋆^(*s*) · *n*(*s*) = *a* ^⋆^(*s*) · *a*(*s*), which is known from the recursive identification. The transfer function of the controller (20) is for this concrete ELM (12):

(23) $$\begin{array}{c} \tilde{Q}\left(s\right)=\frac{q\left(s\right)}{s\cdot \tilde{p}\left(s\right)}=\frac{q_{1}s+q_{0}}{p_{0}\cdot s} \end{array}$$

and parameters *q*_1_, *q*_0_, and *p*_0_ are computed from Diophantine equation (21).

The control synthesis presented above is derived in the continuous-time, but identification and recomputation of the controllers parameters run in discrete-time (*δ*-models). That is why we call this controller* hybrid adaptive controller*.

## 4. Classic Adaptive Control

It is good to show how the nonlinear adaptive control could improve classic adaptive control described, for example, in [10] or [11].

**Figure 11:**
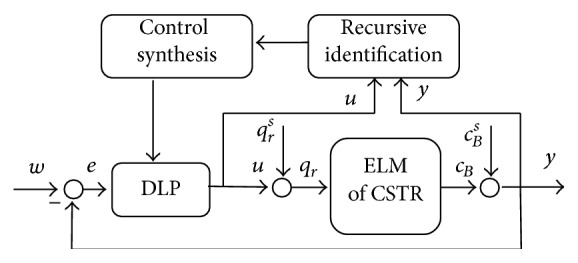
Control scheme of the classic adaptive control.

Let us consider the control configuration displayed in Figure 11 without the SNP part. 

This means that system is controlled only with the use of adaptive controller based on the ELM without the knowledge about static behavior of the system. The design and computation of the controller are the same as what is described in Sections 3.2–3.4. The only difference is that the input variable to the ELM is here *u*(*t*) unlike *u*_0_(*t*) in the nonlinear adaptive control described above.

Results of this control are displayed and commented on in the next section.

## 5. Simulation Experiment

The goal of this last section is to verify proposed classic and nonlinear adaptive controllers by simulations on the mathematical model (1) of the CSTR. The simulations were done for three values of the *α* from (22) which could be understood as a tuning parameter. The sampling period was *T*_*v*_ = 0.1 min, the simulation time was 75 min, and 5 step changes of the reference signal *w*(*t*) were done during this time.

**Figure 12:**
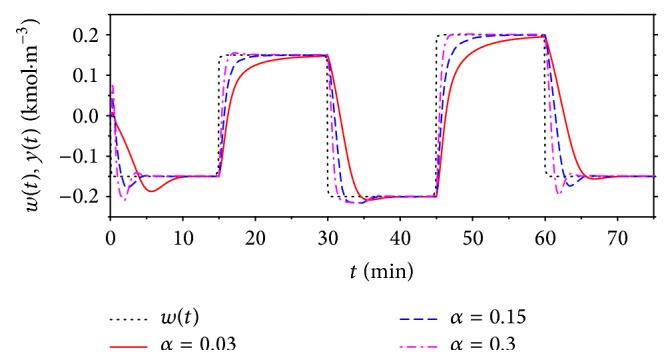
The course of the output variable *y*(*t*) and the reference signal *w*(*t*) for various values of the tuning parameter *α* for nonlinear adaptive control.

**Figure 13:**
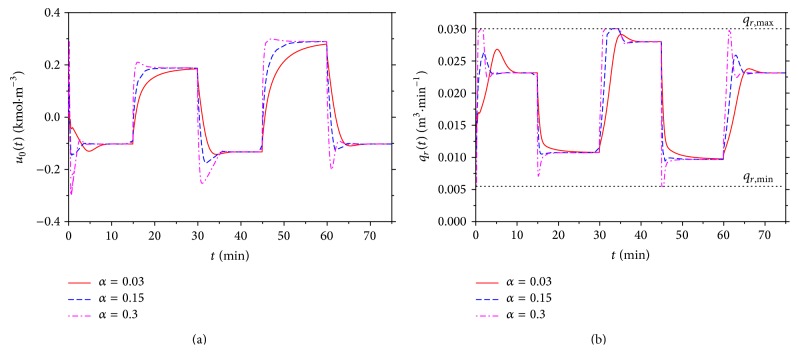
Outputs from the LDP *u*_0_ or various values of *α* (a) and the courses of the computed input variable, *q*_*r*_, to the ELM for various *α* (b).

Figure 12 shows courses of the output variable, *y*(*t*), for various *α* = 0.03, 0.15, and 0.3. It is clear that the increasing value of this parameter results in the quicker output response but overshoots especially for the negative step changes. On the other hand, Figure 13 shows the course of the input variable *u*_0_  (Figure 13(a)) as an output from the DLP which is also input to the SNP.  Figure 13(b) is the course of the volumetric flow rate *q*_*r*_ as an output from the SNP and the input to the mathematical model of CSTR; see schematic representation in Figure 6. We can say that decreasing value of the parameter *α* results in smoother course of both input variables.

**Figure 14:**
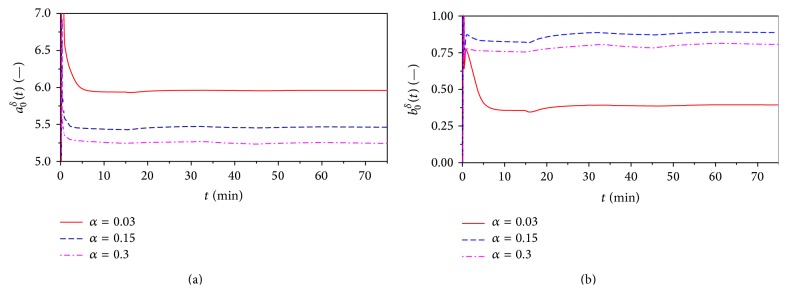
The course of identified parameters *a*_0_^*δ*^ (a) and *b*_0_^*δ*^ (b) for nonlinear adaptive controller.

The course of identified parameters during the control is shown in Figure 14. Graphs show usability of proposed recursive least-squares method with exponential forgetting that is used for online identification of the ELM. The only problem could be found at the very beginning of the control because it needs some initial time to stabilize the parameters as the identification starts from general values of the vector of parameters *θ*_*δ*_ = [0.1 0.1]^*T*^.

**Figure 15:**
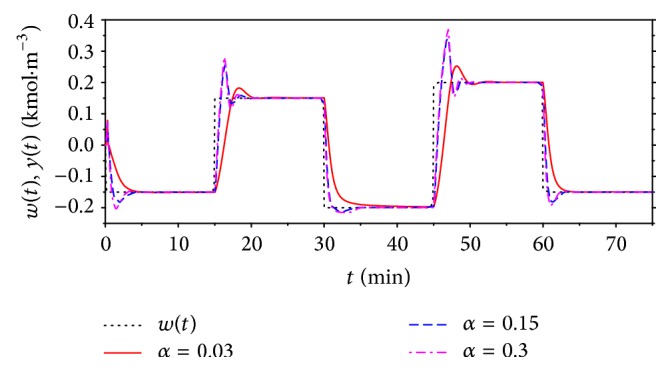
The course of the output variable *y*(*t*) and the reference signal *w*(*t*) for various values of the tuning parameter *α* for classic adaptive control.

**Figure 16:**
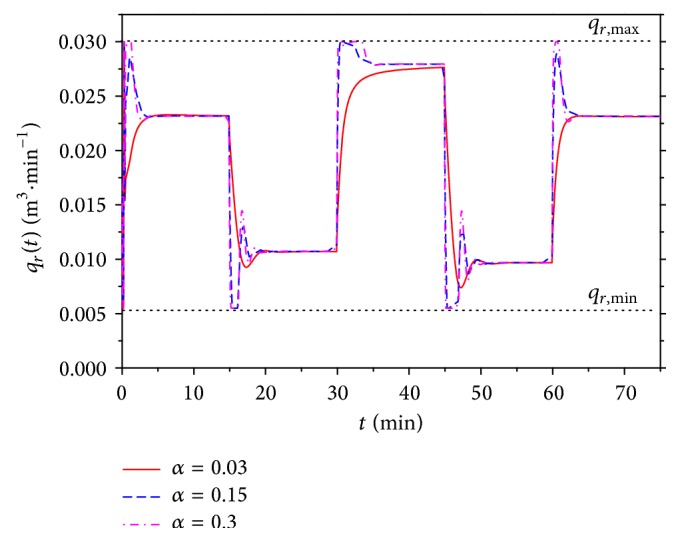
The courses of the computed input variable, *q*_*r*_, to the ELM for various *α* for classic adaptive control.

The task of this contribution was also to show improvement of the nonlinear adaptive approach compared with the classic adaptive control described in Section 4. The simulation studies were done for the same values of the root position *α* = 0.03, 0.15, and 0.3 and results are shown in Figures 15 and 16.

**Figure 17:**
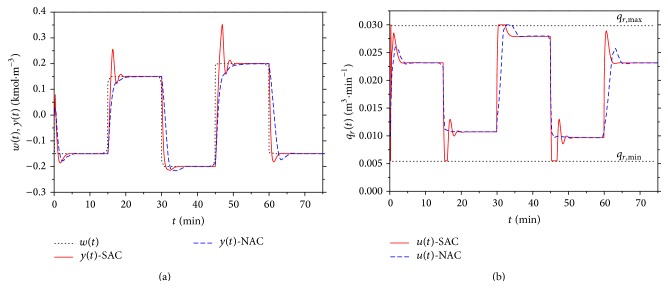
Comparison of resulted courses of output variable *y*(*t*) (a) and input variable *q*_*r*_(*t*) (b) for nonlinear and classic adaptive control for *α* = 0.15.

Compared control results for *α* = 0.15 are shown in Figure 17. Results for both comparisons have shown that nonlinear adaptive control produces better control results especially for the positive changes of the reference signal *w*(*t*). The improvement is evident also for the course of the input value *u*(*t*) which could be also very important from the practical point of view.

## 6. Conclusion

The paper deals with the adaptive control of the CSTR as a typical member of the nonlinear system with lumped parameters. The mathematical model of such system is described by the set of four nonlinear ordinary differential equations and simulation is in this case related to the numerical solution of this set of ODEs. The static and dynamic analysis have shown high nonlinearity of this system which means that controlling of such process with conventional control methods could lead to suboptimal or even very bad control results. The adaptive control is one way to how we can overcome this problem. The adaptive approach here was based on the choice of the delta external linear model of the originally nonlinear system, parameters of which are identified recursively during the control, and the parameters of the controller are also recomputed according to these identified ones. This method satisfies appropriate reaction of the controller to the change of the state of the system or the random disturbance. The control synthesis employs polynomial theory together with the pole-placement method and spectral factorization. These methods satisfy basic control requirements such as stability, reference signal tracking, and disturbance attenuation. The contribution shows also the improvement of this so-called classic adaptive control by the nonlinear theory which is based on the Wiener system where the controller is divided into the dynamic linear part and the static nonlinear part. The dynamic linear part is the same as in classic adaptive control but the static nonlinear part uses simulated or measured steady-state characteristics of the mathematical model to describe the relation between controlled concentration of the product and the change of the reactants volumetric flow rate as an input variable. Both controllers could be tuned by the choice of the parameter *α* as a position of the root in the pole-placement method. Presented results have shown that increasing value of this parameter results in quicker output response but with overshoots for both standard and nonlinear adaptive controllers. Comparison of both controllers with the same settings has shown better control results for nonlinear adaptive control especially for the positive step changes of the reference signal. Although the system has nonlinear behavior, proposed control strategies cope with it well and it could be used also for similar types of systems.

## Figures and Tables

**Table 1:** Fixed parameters of the CSTR.

Name of the parameter	Symbol and value of the parameter
Volume of the reactant	*V*_*r*_ = 0.01 m^−3^
Density of the reactant	ρ_*r*_ = 934.2 kg·m^−3^
Heat capacity of the reactant	*c*_*pr*_ = 3.01 kJ·kg^−1^·K^−1^
Weight of the coolant	*m*_*c*_ = 5 kg
Heat capacity of the coolant	*c*_*pc*_ = 2.0 kJ·kg^−1^·K^−1^
Surface of the cooling jacket	*A*_*r*_ = 0.215 m^2^
Heat transfer coefficient	*U* = 67.2 kJ·min^−1^·m^−2^·K^−1^
Preexponential factor for reaction 1	*k*_01_ = 2.145 · 10^10^ min^−1^
Preexponential factor for reaction 2	*k*_02_ = 2.145·10^10^ min^−1^
Preexponential factor for reaction 3	*k*_03_ = 1.5072·10^8^ min^−1^·kmol^−1^
Activation energy of reaction 1 to *R*	*E*_1_/*R* = 9758.3 K
Activation energy of reaction 2 to *R*	*E*_2_/*R* = 9758.3 K
Activation energy of reaction 3 to *R*	*E*_3_/*R* = 8560 K
Enthalpy of reaction 1	*h*_1_ = −4200 kJ·kmol^−1^
Enthalpy of reaction 2	*h*_2_ = 11000 kJ·kmol^−1^
Enthalpy of reaction 3	*h*_3_ = 41850 kJ·kmol^−1^
Input concentration of compound *A*	*c*_*A*0_ = 5.1 kmol·m^−3^
Input temperature of the reactant	*T*_*r*0_ = 387.05 K
